# Diazeniumdiolate Mediated Nitrosative Stress Alters Nitric Oxide Homeostasis through Intracellular Calcium and S-Glutathionylation of Nitric Oxide Synthetase

**DOI:** 10.1371/journal.pone.0014151

**Published:** 2010-11-30

**Authors:** Yefim Manevich, Danyelle M. Townsend, Steven Hutchens, Kenneth D. Tew

**Affiliations:** 1 Department of Cell and Molecular Pharmacology and Experimental Therapeutics, Medical University of South Carolina, Charleston, South Carolina, United States of America; 2 Department of Pharmaceutical and Biomedical Sciences, Medical University of South Carolina, Charleston, South Carolina, United States of America; Bauer Research Foundation, United States of America

## Abstract

**Background:**

PABA/NO is a diazeniumdiolate that acts as a direct nitrogen monoxide (NO) donor and is in development as an anticancer drug. Its mechanism of action and effect on cells is not yet fully understood.

**Methodology/Principal Findings:**

We used HPLC and mass spectrometry to identify a primary nitroaromatic glutathione metabolite of PABA/NO and used fluorescent assays to characterize drug effects on calcium and NO homeostasis, relating these to endothelial nitric oxide synthase (eNOS) activity. Unexpectedly, the glutathione conjugate was found to be a competitive inhibitor of sarcoplasmic/endoplasmic reticulum Ca^2+^-ATPase (SERCA) presumably at the same site as thapsigargin, increasing intracellular Ca^2+^ release and causing auto-regulation of eNOS through S-glutathionylation.

**Conclusions/Significance:**

The initial direct release of NO after PABA/NO was followed by an eNOS-mediated generation of NO as a consequence of drug-induced increase in Ca^2+^ flux and calmodulin (CaM) activation. PABA/NO has a unique dual mechanism of action with direct intracellular NO generation combined with metabolite driven regulation of eNOS activation.

## Introduction

Endogenous NO is a potent signaling molecule influencing numerous physiological functions. Cellular levels of NO are controlled by several isoforms of nitric oxide synthase (NOS): neuronal (nNOS, NOS1), inducible (iNOS, NOS2), and endothelial (eNOS, NOS3). Each isoform is a product of a distinct gene [1]. Both, nNOS and eNOS, are constitutively expressed and primarily isolated from neurons and endothelial cells, respectively. NO generation by these enzymes is controlled by the elevation of intracellular Ca^2+^ and the consequent activation of calmodulin (CaM). iNOS is not constitutively expressed and is not calcium-dependent. Despite its physiological functions, high levels of intracellular NO are toxic and provide a translational opportunity to induce cytotoxicity in tumor cells [2]. This led to the development of a class of anticancer agents selectively activated in tumors by glutathione S-transferase pi (GSTP) to liberate toxic levels of NO [3]. The contribution of NOS to the cytotoxic effects of these agents has not been explored and is the focus of these studies.

Para-amino-benzoic acid (PABA) has been tested as a radioprotector [4] and PABA/NO (O^2^-(2,4-dinitro-5-[4-(N-methylamino)benzoyloxy]phenyl}1-(N,N-dimethylamino)diazen-1-ium-1,2-diolate) is an anticancer prodrug with antitumor activity *in vitro* and in human ovarian cancer xenograft mouse models [5], [6]. PABA/NO has N-methyl-p-aminobenzoic acid bound via its carboxyl oxygen as a 5-substituent of the 2,4-dinitrophenyl ring [3]. PABA/NO belongs to the O2-aryl diazeniumdiolates (O2ADs) — electrophiles shown to transfer their aryl groups to the attacking nucleophiles with a simultaneous production of ions that spontaneously release NO at a physiological pH [7]. In the presence of glutathione (GSH), PABA/NO becomes activated (spontaneously or through the glutathione S-transferase pi (GSTP)-mediated catalysis) and results in the formation of a Meisenheimer-complex intermediate, where subsequently the leaving group of the reaction generates two moles of NO [7]. As a consequence, elevated NO levels lead to cytotoxic effects by forming reactive nitrogen/oxygen intermediates. PABA/NO-induced nitrosative stress results in limited levels of protein nitrosylation/nitration and high levels of S-glutathionylation, and these are associated with cytotoxicity in human promyelocytic leukemia (HL60) cells [6].

S-glutathionylation is an oxidative post-translational modification of low pKa cysteine residues in target proteins. The forward rate of the S-glutathionylation reaction is regulated by GSTP [8], [9], [10], [11], while the reverse rate is regulated by a number of redox sensitive proteins, including glutaredoxin [12], thioredoxin and sulfiredoxin [13], [14]. Proteins affected by S-glutathionylation include ion channels such as a Ca^2+^-release/ryanodine receptor channel (RyR) and a phosphorylation/ATP-dependent chloride channel that modulates salt and water transport in the lung and gut [15], [16], [17]. Regulatory effects of S-glutathionylation have also been described for the SERCA, [18]. Following peroxynitrite treatment, SERCA is S-glutathionylated at Cys674, both *in vitro* and in intact cells or arteries [18], [19]. This modification activates SERCA, resulting in a decrease of cytosolic Ca^2+^.

Alterations in intracellular Ca^2+^ can be associated with its influx from the extracellular space, as well as by its release from intracellular stores (ER, SR, mitochondria etc). Increased intracellular concentrations of free Ca^2+^ influence a number of cellular processes that include proliferation, contractility and secretion [20], [21]. Plasma membranes have an initially low permeability to Ca^2+^, with active Ca^2+^ uptake occurring against an electrochemical gradient. This process is mediated by Ca^2+^ -ATPases contained in both plasma and organelle membranes of intracellular Ca^2+^ stores. The overall result is that intracellular Ca^2+^ is maintained at low levels.

We have focused the present study on understanding how PABA/NO metabolism may influence NO homeostasis both directly and indirectly through altering intracellular Ca^2+^ and NOS activity.

## Results

### Kinetic analysis of PABA/NO-derived NO generation in HL60 cells as compare to that of the short- and long-lived standard NO generators

**Figure 1:**
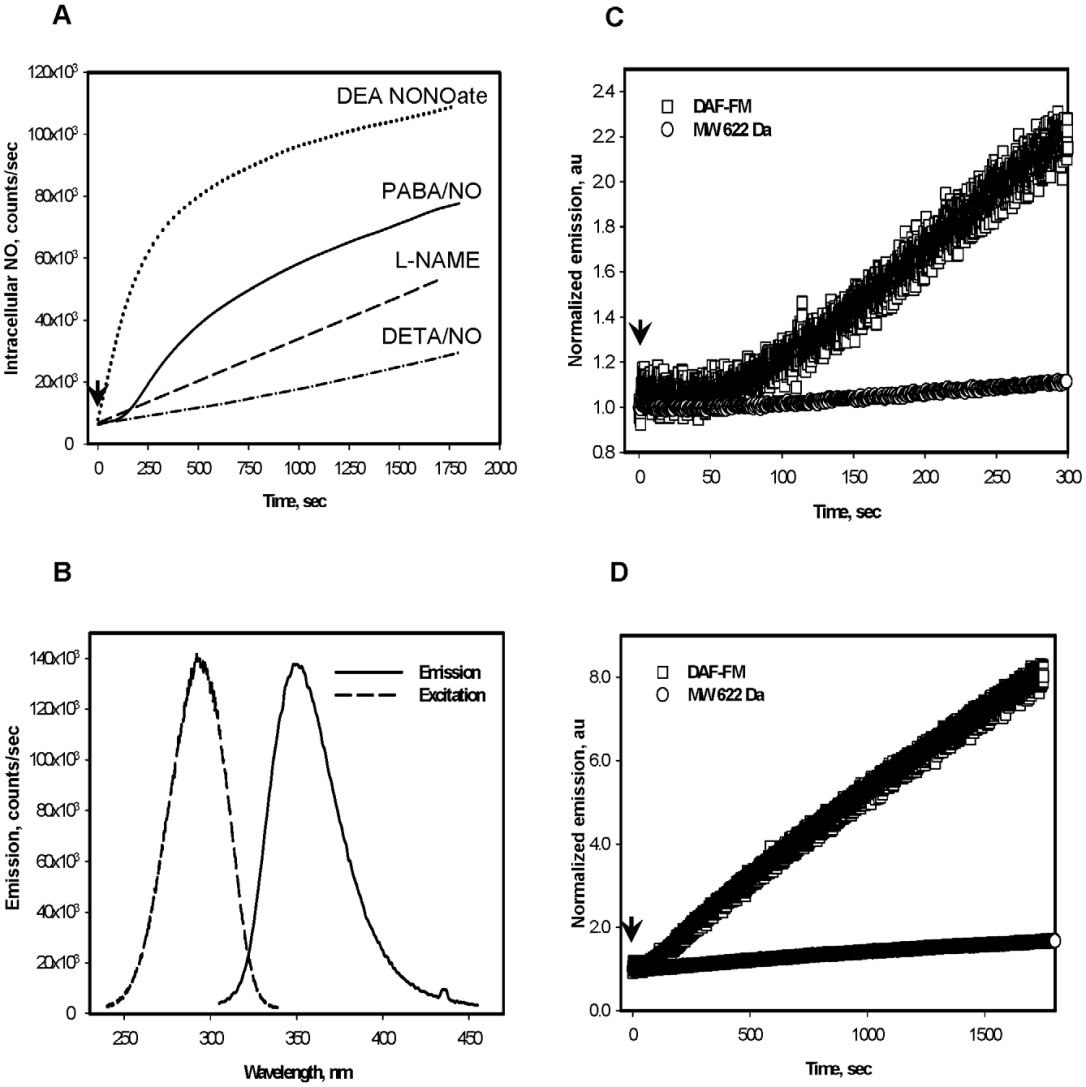
PABA/NO-induced NO and fluorescent derivative generation kinetics in HL60 cells. A) PABA/NO (25 µM) addition to HL60 cells corresponds with an S-shaped generation of NO, becoming linear after cells are pretreated with 50 nM L-NAME for 30 min prior to PABA/NO (100 and 200 nM of L-NAME addition did not show any difference). DETA/NO and DEA NONOate (25 µM) addition to HL60 cells result in linear NO generation kinetics. B) Emission and excitation spectra of a purified fluorescent nitro-aromatic product (MW 622 Da) C) and D) Short and long exposure of HL60 cells to 25 µM PABA/NO produces linear kinetics for the fluorescent nitro-aromatic product (MW 622 Da) and S-shaped kinetics for NO generation. Data are: trace smoothed with Sigma Plot 10 software (panel A) and original traces (Panels B, C and D) representative of three independent experiments. Arrows indicate addition of drug.

Figure 1 shows the intracellular generation of NO in HL60 cells exposed to either PABA/NO or diethylenetriamine/nitric oxide adduct (DETA/NO). The kinetics of NO release after PABA/NO are shown in Fig. 1, panel A. After an early lag phase, the curve becomes S shaped. This becomes linear (Fig. 1, panel A) after pretreatment of cells with the NOS inhibitor - *N^ω^*-nitro-L-arginine methyl ester (L-NAME, [22]). At equimolar doses, release of NO from DETA/NO was linear, but lower than PABA/NO, and from diethyleneamine NONOate (DEA NONO-ate) initially was linear, but higher than PABA/NO (Fig. 1, panel A), and both were insensitive to L-NAME treatment (data not shown). PABA-NO-mediated NO generation both *in vitro* and *in vivo* coincided with generation of a fluorescent product (Fig. 1, panel B). The kinetics of NO generation shortly (∼300 s) after PABA/NO reflected a ∼2∶1 molar ratio to stable fluorescent product (Fig. 1, panel C). After longer incubations (Fig. 1, panel D) a ∼4∶1 molar ratio indicated an accelerated NO production compared to levels expected merely from PABA/NO decomposition.

### Comparative analysis of Ca^2+^ and NO fluxes initiated by PABA/NO addition to HL60 cells and proportionality of NO generation to eNOS expression

**Figure 2:**
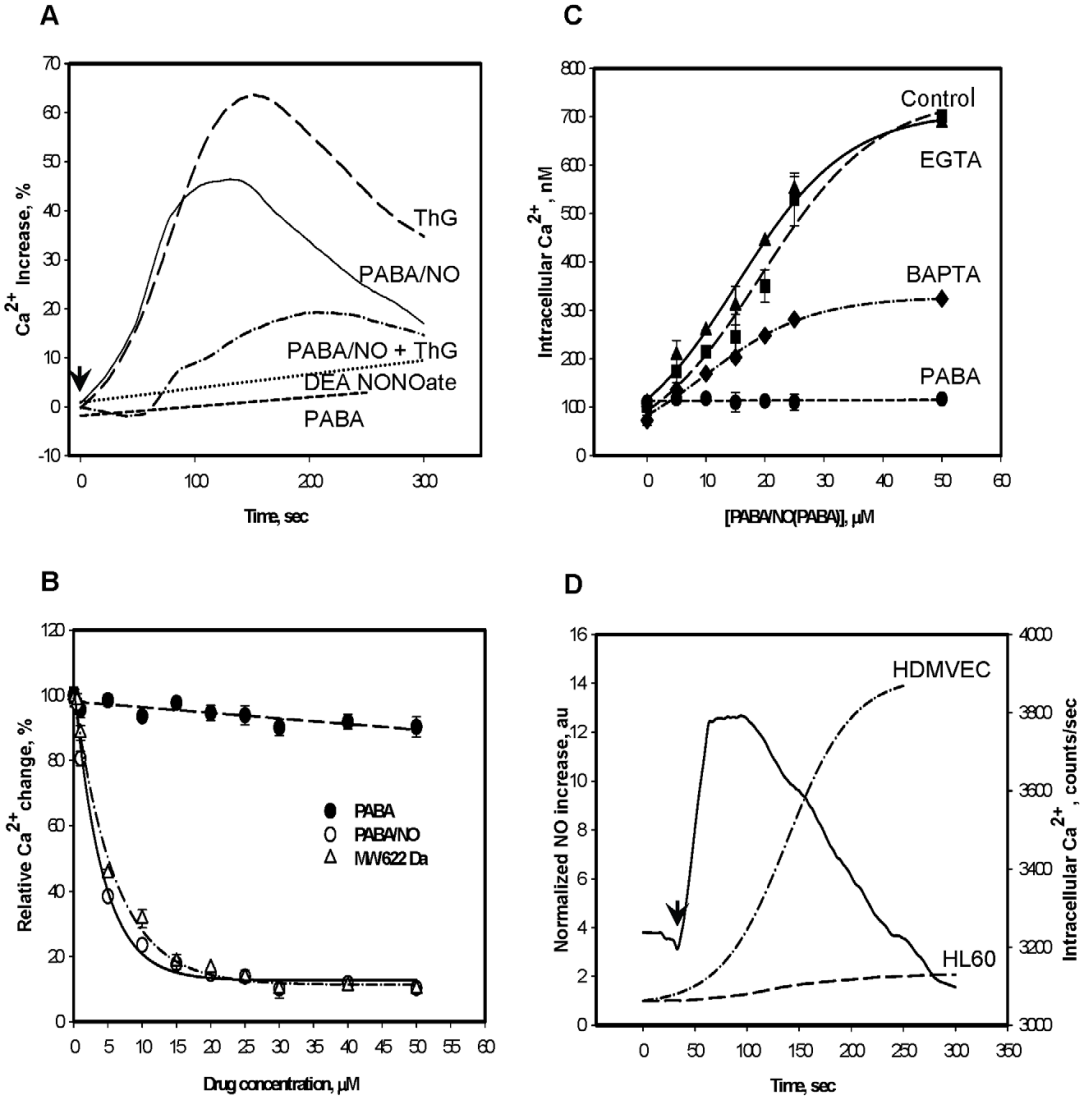
PABA/NO treatment of HL60 cells results in temporal and dose-dependent increases in intracellular Ca^2+^. A) Time-dependent increases were assessed following treatment with 20 nM ThG; 25 µM PABA/NO; 25 µM PABA; 20 nM ThG after pretreatment with PABA/NO (15 µM, 30 min); 25 µM DEA NONOate B) Competitive inhibition of ThG (100 nM) - mediated increases in intracellular Ca^2+^ by PABA/NO, purified homogeneous nitro-aromatic product (MW 622 Da), or PABA. C) Dose-dependent increases in Ca^2+^ following PABA/NO (Control) or PABA were measured; in the presence of the intracellular Ca^2+^ chelator BAPTA-AM (5 µM); or in the presence of the extracellular Ca^2+^ chelator, EGTA (5 mM, long dashedline). The fluorescence measurements were recalculated as actual intracellular Ca^2+^ concentrations (see Materials and Methods). D) Kinetics of intracellular NO (left Y-axis) and Ca^2+^ (right Y-axis, solid line) generation after PABA/NO (15 µM) addition. Data are the average representative traces (A and D) or mean ± SE (B, C) for 3 independent experiments.

The observed lag phase implied a response to an accumulation of signaling molecule. Thus, Fig. 2 shows the time- and dose- dependent calcium mobilization in HL60 cells. Treatment of HL60 cells with 25 µM PABA/NO resulted in a time-dependent bell-shaped increase of intracellular Ca^2+^ with levels increasing proportionally with drug-treatment. Thapsigargin (ThG) mobilizes Ca^2+^ through the inhibition of SERCA and was used as a positive control. The Ca^2+^ increases initiated by ThG (Fig. 2, panel A) were similar to those for PABA/NO. Pre-incubation of HL60 cells with PABA/NO (15 µM) substantially diminished those increases in intracellular Ca^2+^ caused by the addition of 20 nM ThG (Fig. 2, panel A). Titration of HL60 cells with an increased amount of PABA/NO gradually decreased ThG-mediated Ca^2+^ fluxes with an IC_50_ of ∼3 µM (Fig. 2, panel B). Similar titration of cells with purified homogeneous PABA/NO-GSH adduct (MW∼622 Da) was close to that of PABA/NO (IC_50_ ∼5 µM; Fig. 2, panel B). These results are consistent with an intracellular store acting as a source of the released Ca^2+^. Calibration of Fluo-3 fluorescence emission [23] permitted calculation of an actual intracellular Ca^2+^ concentrations in HL60 cells of 110±12.0 nM. Prior chelation of extracellular Ca^2+^ with EGTA (5 mM) minimally affected the PABA/NO-initiated increases in intracellular Ca^2+^, while chelating intracellular Ca^2+^ with BAPTA (6 µM) substantially decreased the PABA/NO-mediated mobilization of Ca^2+^ (Fig. 2, panel C) similar to that for the ThG-mediated Ca^2+^-fluxes (data not shown). Interestingly, treatment of HL60 cells with PABA or DEA NONO-ate (instead of PABA/NO) did not have any significant effect on Ca^2+^ fluxes (Fig. 2, panels A and C). These results implied that PABA/NO-GSH adduct (MW ∼622 Da) is most likely responsible for this specific effect. Plotting the temporal events of Ca^2+^ mobilization and NO generation in HL60 cells after PABA/NO treatment showed that Ca^2+^ increases precede those for NO (Fig. 2, panel D), suggesting Ca^2+^-mediated the NO generation. Although ∼8-fold higher, a similar pattern of NO generation was also seen in human dermal microvascular endothelial cells (HDMVEC, Fig. 2, panel D).

These data suggested a role for Ca^2+^-dependent NO generation following PABA/NO addition. In HL60 cells, NO homeostasis is controlled by the expression of both iNOS (NOS2, calcium independent, [24]) and eNOS (NOS3, calcium-dependent). Based on immunoblot analysis, nNOS (NOS1) was absent from HL60 cells (data not shown).

### siRNA-mediated “knock down” of eNOS in HL60 cells

**Figure 3:**
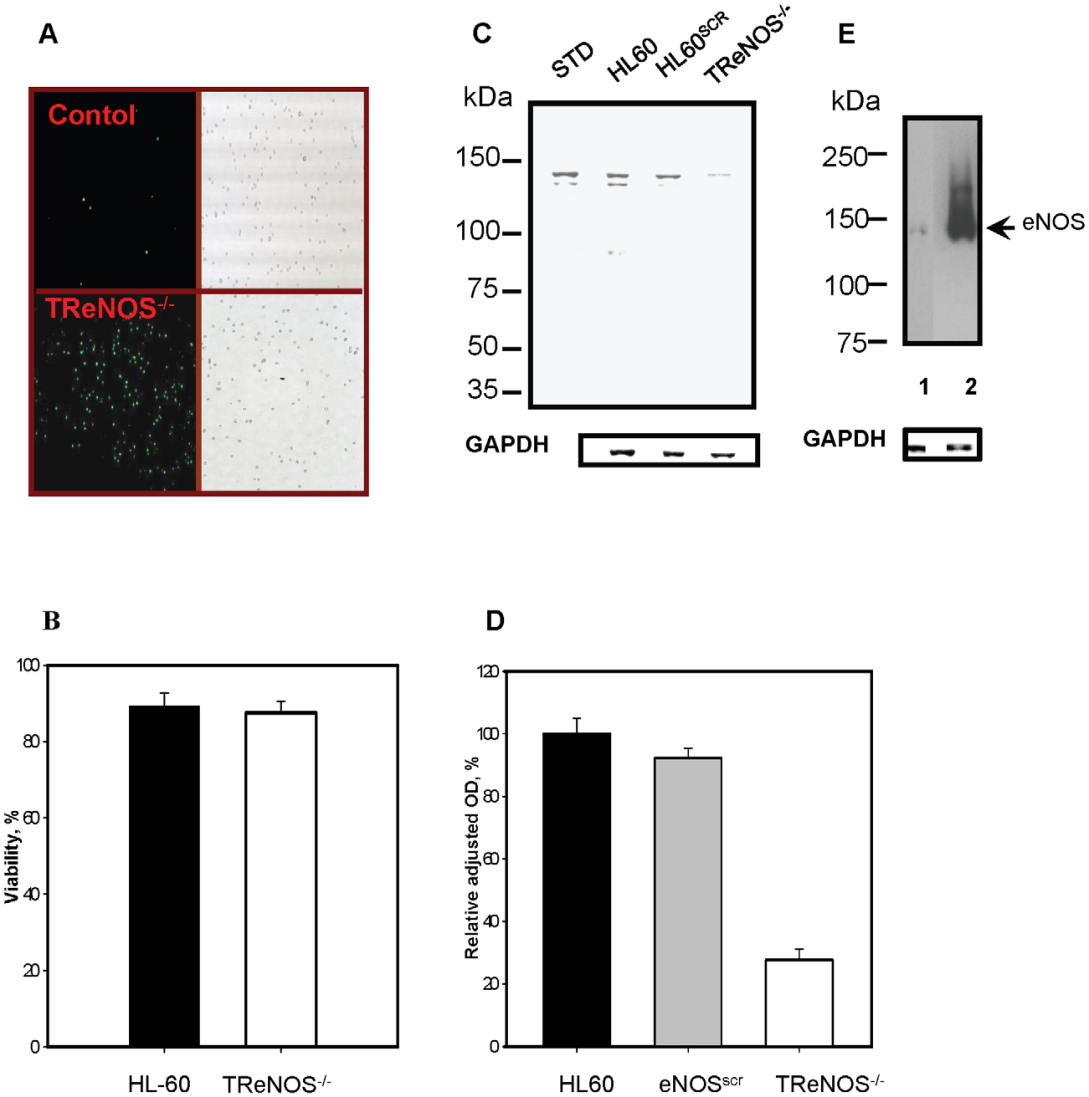
The effect of eNOS suppression in HL60 cells after siRNA transfection. A) Fluorescent and DIC images of cells before (upper panels) and after (lower panels) transfection with FITC-labeled siRNA. B) Viability of mock (black column) and eNOS siRNA transfected cells (white column) detected by trypan blue exclusion. C) Immunoblot analysis of cell lysates (∼100 µg of total protein/lane) compared to standard (STD, Cayman) with eNOS detection. D) Immunoblot (panel C) quantification. E) Immunoblot analysis of eNOS in HL60 cells (1) and in HDMVEC (2) lysates (∼20 µg of total protein/lane); Data in B) and D) represent mean ± SE of 3 independent experiments. Loading controls with GAPDH detection on the same blot are presented.

To determine how PABA/NO treatment might influence NO homeostasis, we transiently suppressed eNOS expression with siRNA. To monitor the transfection efficiency, we used FITC-labeled siRNA. Our results show that the transfection efficiency of eNOS siRNA in HL60 cells was ∼80% (Fig. 3, panel A). Suppression of eNOS did not alter cell viability (Fig. 3, panel B), but resulted in ∼80% decrease of eNOS protein expression (Fig. 3, panel C-D). By comparative immunostaining, the level of eNOS was found to be ∼10-fold higher in HDMVEC when compared to HL60 cells (Fig. 3 panel E).

### Effect of eNOS “knock down” in HL60 cells on the PABA/NO-mediated NO and Ca^2+^ fluxes

**Figure 4:**
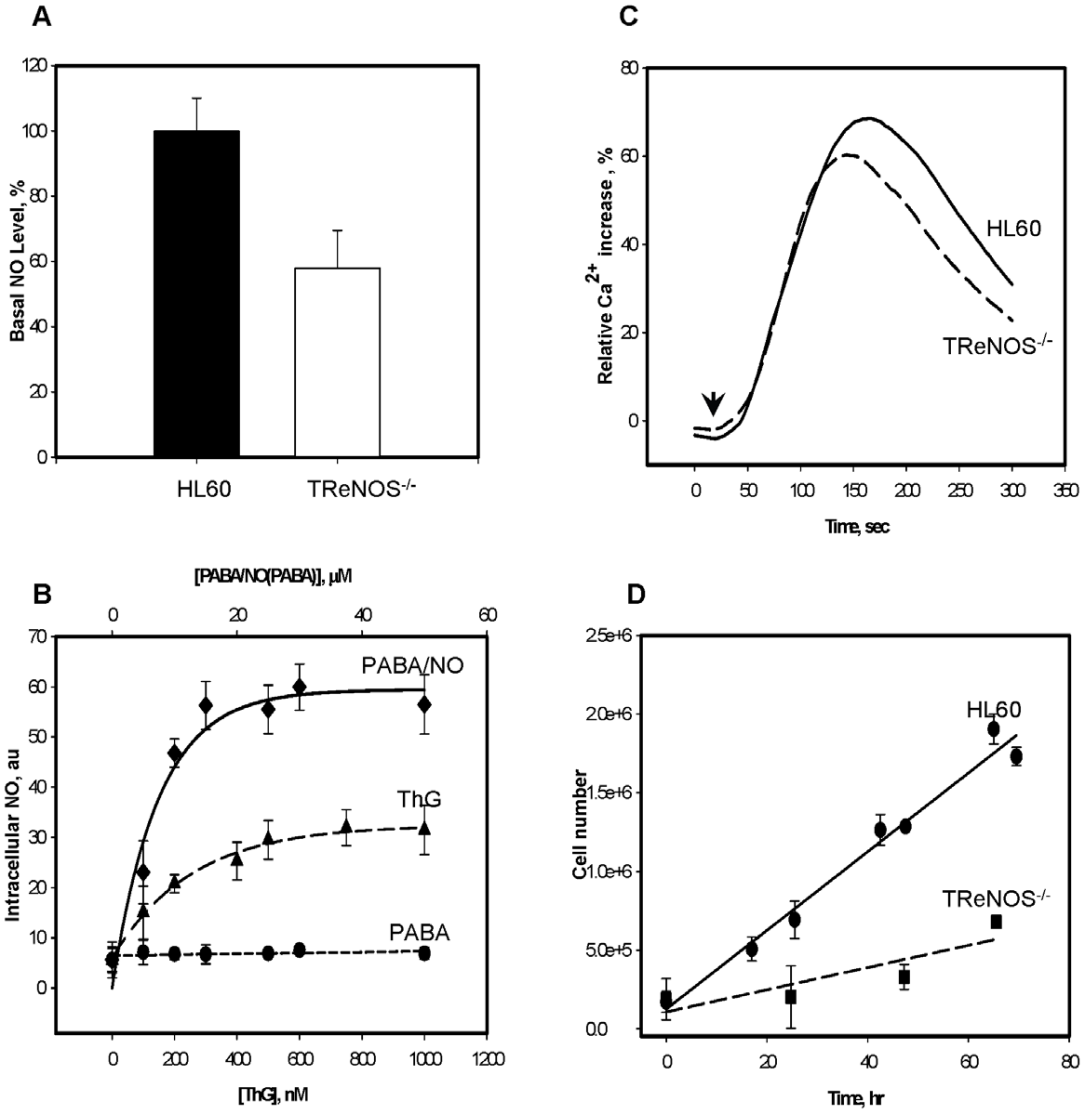
eNOS depletion alters basal and PABA/NO mediated NO generation and cell growth. A) HL60 and TReNOS^−/−^ cells labeled with DAF-FM to measure total basal NO levels. B). NO generation in HL60 cells following PABA/NO, PABA, or ThG titration. C) Kinetics of intracellular Ca^2+^ increase in TReNOS^−/−^ after PABA/NO (25 µM) or ThG (20 nM) addition. D) Growth curves for HL60 and TReNOS^−/−^ cells. Data represent: mean ± SE (panels A and B) for 3 independent experiments; representative traces of 3 independent experiments (panel C); and average ± variance for 2 independent experiments (panel D).

Basal levels of NO in the eNOS siRNA transfected cells (TReNOS^−/−^) were decreased by ∼50%, (Fig. 4, panel A). The siRNA depletion of eNOS levels did not impact the intracellular Ca^2+^ response to PABA/NO or ThG (Fig. 4, panel C), which remained similar to that of the control cells (Fig. 2, panel A). NO generation following either 0–50 µM PABA/NO or 0–1,000 nM ThG treatments was statistically different with the EC_50_ values of ∼5 µM and ∼85 nM, respectively. PABA did not affect NO generation in HL60 cells (Fig. 4, panel B). These data are compatible with an ∼50% effect of Ca^2+^-dependent NO generation in total response of HL60 cells to PABA/NO. The eNOS suppression in HL60 cells was accompanied by a slowing of cell growth rate (Fig. 4, panel D), indicating a link between NO metabolism and the cell proliferation pathways.

### Effect of calmodulin on the PABA/NO-derived NO generation

**Figure 5:**
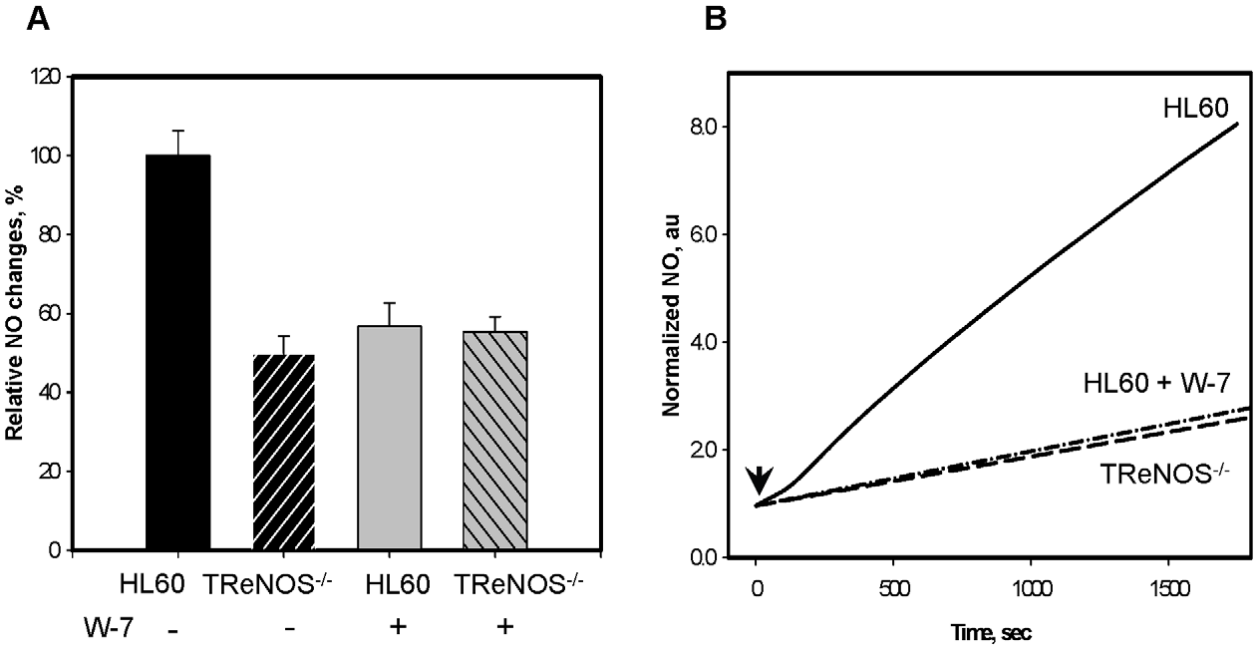
Inhibition of calmodulin diminishes NO generation following PABA/NO treatment. A) Basal NO levels in HL60 and TReNOS^−/−^ cells before and after incubation with 50 nM of the specific CaM-inhibitor W-7. B) Kinetics of NO generation in HL60, HL60 cells after preincubation (30 min) with 50 nM of W-7, and in TReNOS^−/−^ cells following PABA/NO (25 µM) addition. Data represent: mean ± SE (panel A) and representative traces (panel B) of 3 independent experiments.

CaM is a key regulator in eNOS activation. HL60 and TReNOS^−/−^ cells were treated with the CaM inhibitor W-7 prior to PABA/NO treatment. The addition of W-7 to HL60 cells resulted in ∼50% decrease in NO levels when compared to the untreated cells (Fig. 5, panel A). In contrast, W-7 to did not affect intracellular NO levels in TReNOS^−/−^ cells (Fig. 5, panel A). Inhibition of CaM in HL60 cells substantially diminished the NO response similar to that in TReNOS^−/−^ cells following PABA/NO addition (Fig. 5, panel B). These data are compatible with a partial eNOS-mediated NO response of HL60 cells to PABA/NO in addition to known spontaneous and/or GSTP-mediated GSH nucleophilic attack on this prodrug [5].

### Estimated basal and PABA/NO-mediated levels of NO in HL60 cells

To estimate basal levels of NO in HL60 cells, quantitative fluorescence measurements of these cells were compared with human umbilical vein endothelial cells (HUVEC), which contain ∼20 nM NO (see Materials and Methods). Our calculations estimated NO concentrations at ∼1.2 nM in HL60 cells and ∼14.0 nM in HDMVEC (Table 1). Addition of PABA/NO (5 µM) caused increases of NO levels to ∼2.6 nM in HL60 and to ∼182.0 nM in HDMVEC.

**Table 1:** Estimated basal and PABA/NO-induced intracellular NO levels.

*Cells*	*N*	*DAF-FM emission, counts/sec*	*[NO], nM*
HUVEC	5	1.062±0.1164	20.0
HDMVEC	5	0.738±0.1604	14.0±3.0
HDVEC+PABA/NO (5 µM)	5	9.594±0.9124	182.0±17.3
HL60	6	0.0624±0.00525	1.2±0.1
HL60+PABA/NO (5 µM)	6	0.1354±0.01175	2.6±0.2
HL60+PABA/NO (25 µM)	6	0.4992±0.0487	9.6±0.3

Data represents mean±SEM for indicated number (N) of experiments.

### Effect of PABA/NO treatment on eNOS glutathionylation in HL60 and HDMVE cells

**Figure 6:**
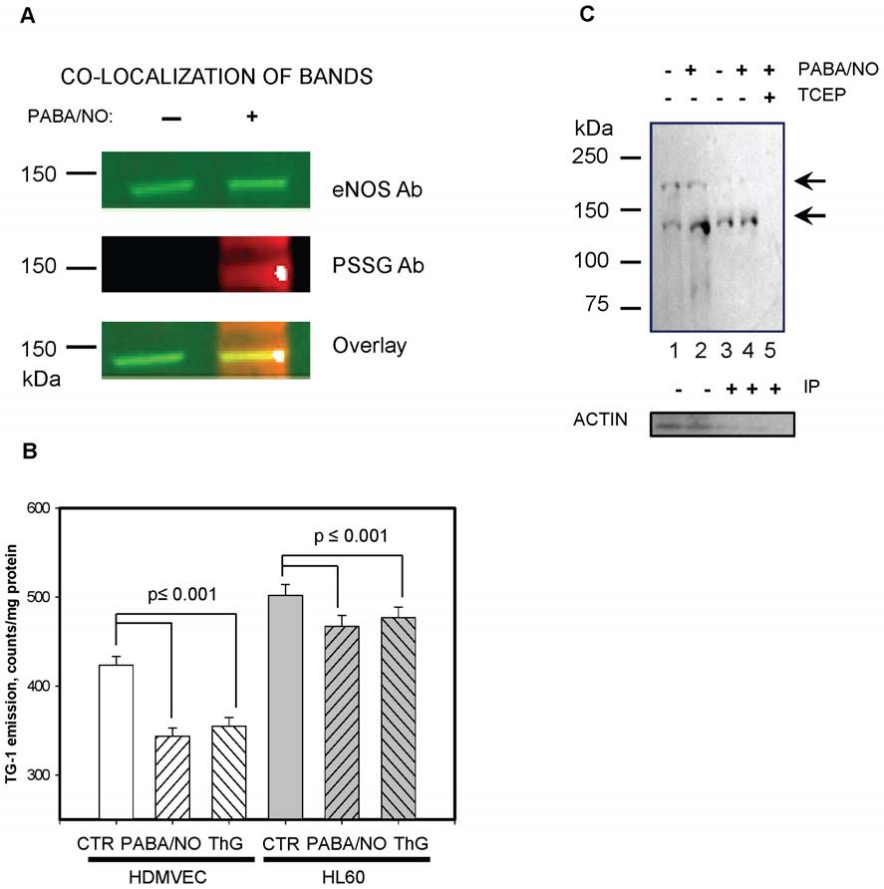
PABA/NO-mediated S-glutathionylation of eNOS in HL60 and HDMVE cells. A) Control and PABA/NO-treated (25 µM, 30 min) HL60 cell lysates (∼100 µg protein/lane) resolved by non-reducing SDS-PAGE and evaluated by immunoblot with eNOS polyclonal primary antibodies and PSSG monoclonal primary antibodies and detected simultaneously with both: anti-mouse (red) and anti-rabbit (green) fluorescent secondary antibodies. B) Fluorescent detection (TG-1) of cytosolic protein sulfhydryls in HDMVE and in HL60 cells before (CTR) and after: PABA/NO (20 µM, 30 min) or ThG (200 nM, 30 min) addition. C) Immunoprecipitation of eNOS from HDMVEC with S-glutathionylated protein antibody. The blot was developed with the eNOS antibodies. Lanes are: 1- control HDMVEC lysate; 2- PABA/NO-treated (20 µM, 30 min) cell lysate; 3- IP of control cell lysate; 4- IP of PABA/NO-treated cell lysate; 5 -IP of PABA/NO-treated cell lysate after TCEP treatment. Arrows indicate low and high molecular weight eNOS bands. Loading controls are actin immunostaining. Data are: representative blots (panels A, C) and mean ± SD of 3 independent experiments, statistical significance p≤0.001.

PABA/NO- mediated NO generation in HL60 cells became linear after an incubation for ∼600 s (Fig. 1, panel A). This could be associated with an auto-regulation of eNOS by NO in HL60 cells. We hypothesized that eNOS S-glutathionylation may be involved in this inhibition. After incubating cells with 25 µM PABA/NO for 30 min, co-staining of eNOS with the S-glutathionylated protein antibodies in HL60 cells was detected (Fig. 6, panel A). The expression of eNOS was substantially greater in HDMVEC than in HL60 cells. Fig. 6 shows the effects of PABA/NO on the S-glutathionylation of monomeric (∼137 kDa) and the higher molecular weight band (∼230 kDa) of eNOS under non-reducing conditions. The reduction of the disulfide bonds with TCEP caused the high molecular weight band to disappear (data not shown), suggesting that it could represent disulfide bond formation between denatured monomers during sample boiling in SDS. PABA/NO treatment of HDMVEC resulted in a decrease of the high molecular weight band and in a consequent increase of eNOS monomer, perhaps as a consequence of sulfhydryl modification through S-nitrosylation or S-glutathionylation. In HDMVEC, the eNOS monomer was immunoprecipitated with the anti-glutathionylated protein antibody and there was a quantitative increase following PABA/NO treatment (Fig. 6, panel C), a result consistent with our hypothesis. In addition, it is known that both PABA/NO and ThG cause S-glutathionylation of multiple proteins in HL60 cells [8], [25]. Thus, we analyzed the total cellular protein sulfhydryls at steady state and following drug treatment in both HL60 and HDMVEC (Fig. 6, panel B). PABA/NO caused a ∼6% decrease in cytosolic protein sulhydryls in HL60 and ∼15% decrease in HDMVEC versus the controls. The latter indicates sulfhydryl modification upon PABA/NO treatment. Basal protein sulfhydryl levels in HL60 cells were higher than in HDMVEC (inversely proportional to eNOS level in these cells). The ThG effect was not statistically different from that of PABA/NO. The addition of TCEP resulted in disappearance of all bands, indicating IP specificity for S-glutathionylated proteins.

### Plasma membrane associated effects of PABA/NO treatment in HL60 cells

**Figure 7:**
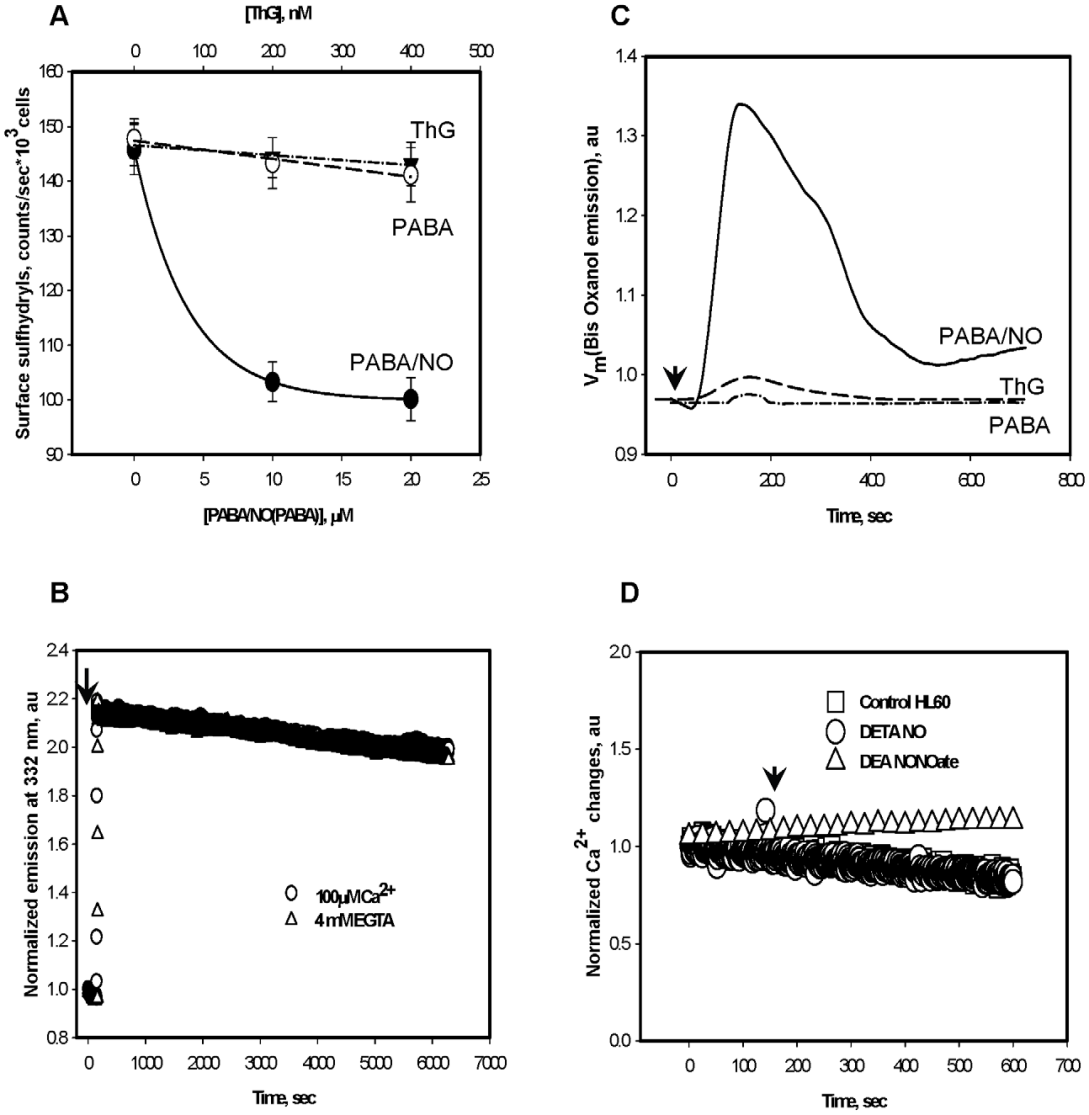
PABA/NO effect on surface protein thiols and plasma trans-membrane potential in HL60 cells and controls for PABA, DETA/NO and DEA NONOate effects. A) Fluorescent detection (TG-1) of dose-dependent HL60 surface protein sulfhydryls level after PABA/NO, PABA and ThG addition. B). PABA (50 µM) addition to HL60 cells (with or without extracellular Ca^2+^) did not initiate generation of fluorescent metabolite (MW 622). C) Plasma trans-membrane potential (V_m_) recorded as Bis-Oxanol emission at 520 nm after addition of 20 nM ThG, 25 µM PABA or 25 µM PABA/NO in HL60 cells. D) DETA/NO and DEA NONOate (25 µM) addition to HL60 cells did not initiate Ca^2+^ fluxes. Data represent: mean ± SE (panel A), representative traces averaged and smoothed with Sigma Plot 10.0 software (panel C), and original representative traces (panel B and D) of 3 independent experiments.

PABA/NO is poorly soluble and relatively unstable in water. This may result in decomposition-mediated NO generation. NO is easily diffusible through plasma membranes and it is difficult to discriminate between processes of intracellular or extracellular generation. HL60 cell surface protein sulfhydryl modifications were monitored after PABA/NO or ThG using TG-1. A dose-dependent but saturable decrease of cell surface sulfhydryls was detected after the PABA/NO addition (Fig. 7, panel A). Subsequent TCEP addition to PABA/NO treated HL60 cells did not reverse the drug-mediated sulfhydryl modifications (data not shown) indicating an absence of disulfide bonds and perhaps, corresponding with cell surface protein thiol nitrosylation. In contrast, neither PABA nor ThG directly yields NO and neither affected the surface protein-thiol status (Fig.7, panel A).

NO synthesis and calcium cycling are known to modulate membrane polarization. We evaluated the plasma membrane potential (***V_m_***) in HL60 cells following PABA/NO or ThG treatment. Immediate hyperpolarization of the plasma membrane occurred after either PABA/NO or ThG treatment with a subsequent relatively slow (∼200–250 sec) depolarization. The effect for PABA/NO was ∼10 times greater when compared to ThG and showed a slower rate of depolarization, suggesting a multistep process (Fig. 7, panel C). PABA addition resulted in negligible effects on plasma membrane potential indicating the involvement of the NO-generating capacity of PABA/NO, its initiation of Ca^2+^ fluxes, and secondary eNOS-mediated intracellular NO production. Our data also show that addition of PABA (fluorescent Ex.∼283 nm, Em. ∼332 nm, 25 µM) to HL60 cells did not initiate any changes in its fluorescence (Fig. 7, panel B). In addition, treatment of HL60 cells with 25 µM of DETA/NO or DEA NONO-ate did not induce Ca^2+^ fluxes (Fig. 7, panel D) regardless of its capacity to generate NO (Fig. 1, panel A). These results indicate that NO itself does not directly mediate Ca^2+^ fluxes in HL60 cells.

### PABA/NO metabolism in HL60 cells

**Figure 8:**
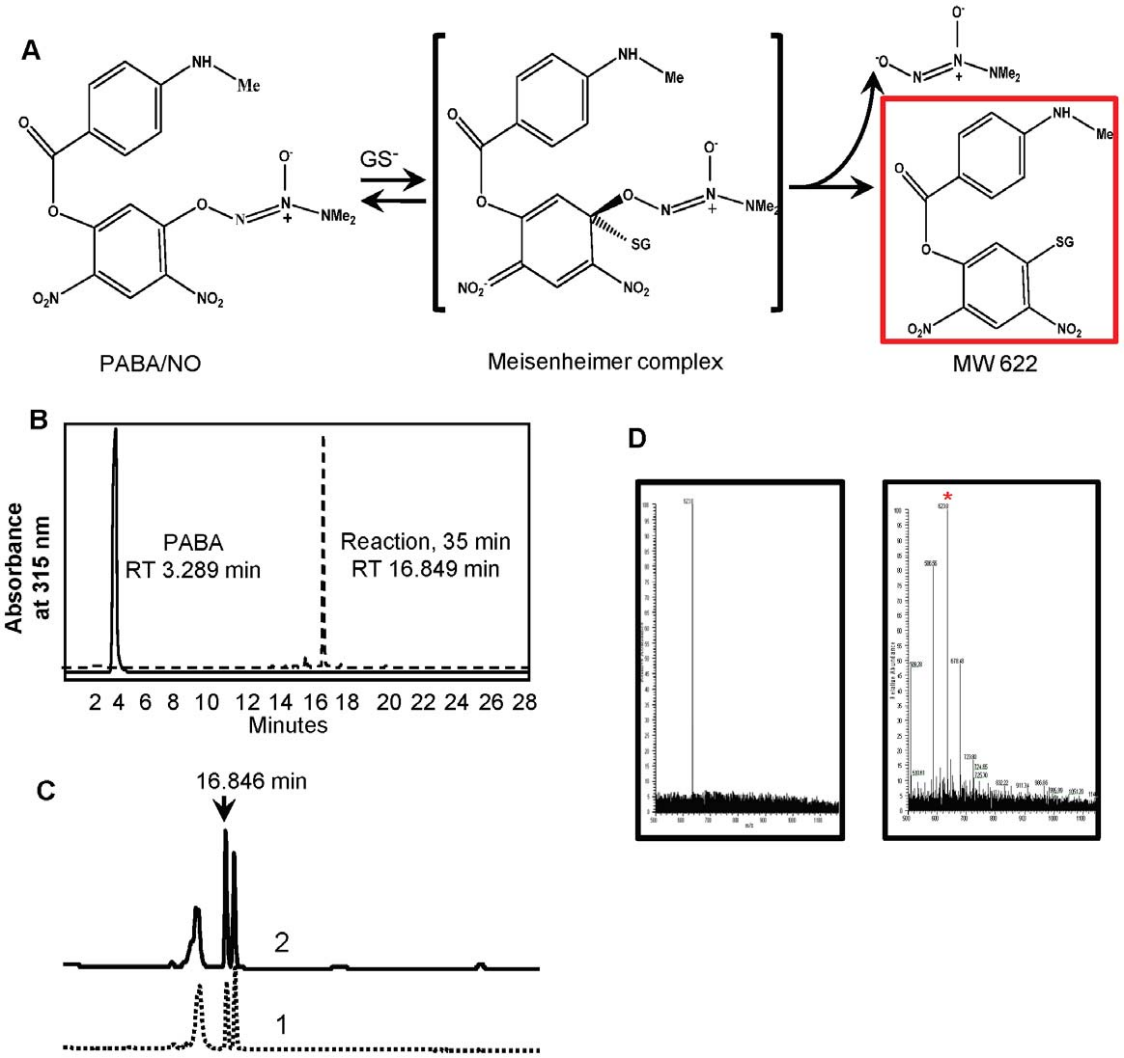
Reaction of PABA/NO with GSH *in vitro* and in HL60 cells. A) Scheme of nitro-aromatic PABA/NO-GSH adduct (MW 622 Da) generation. B) HPLC analysis of PABA/NO-GSH reaction *in vitro* and detection of PABA standard under the same conditions. C) HPLC analysis of HL60 lysate after incubation with 50 µM PABA/NO (1) and after “spike” with the purified nitro-aromatic adduct (MW 622 Da, 2). D) ESI MS analysis of purified from *in vitro* reaction nitro-aromatic PABA/NO-GSH adduct (m/z 623.8, left panel) and HL60 cells lysate after their incubation with purified nitro-aromatic adduct (m/z 623.8) product (right panel, indicated with asterisk).

Fig. 8 shows that either under *in vitro* or *in vivo* conditions, the primary metabolite of PABA/NO has a single HPLC peak with a retention time of 16.84 min. This was characterized further by spiking the sample with purified from *in vitro* reaction metabolite, showing co-mobility of the mixture (Fig. 8 panel C). The molecular mass of this purified product was assessed through ESI MS analysis and m/z was found to be 623.8 (>97% homogeneity, Fig. 8, panel D, left panel). Incubation of HL60 cells with this purified product resulted in its detection in cytosole (Fig. 8, panel D, right panel). Although the data shown are from the cytosolic fraction of HL60 cells, approximately 90% of this glutathione-conjugated metabolite was found in the pellet fraction, implying a possible association with organelles.

## Discussion

The focus of our present study was to investigate the influence of PABA/NO on the dynamics of intracellular Ca^2+^ and NO homeostasis. The data support a role for eNOS in potentiating the direct nitrosative stress caused by PABA/NO decomposition. The effects of PABA/NO on the kinetics of the intracellular Ca^2+^ increases are similar to those for ThG and imply a possible shared target such as SERCA, responsible for release of Ca^2+^ from intracellular stores. This concept is supported by our data suggesting that mobilization of cytosolic Ca^2+^ is independent of extracellular Ca^2+^. These data and the bell-shaped kinetics suggest that PABA/NO and ThG may have similar site(s) of action but with distinct affinities. A direct comparison of ThG and PABA/NO shows that pre-incubation of HL60 cells with PABA/NO substantially diminishes the effects of ThG with ID_50_ of ∼3 µM. ThG is a known SERCA antagonist, which causes Ca^2+^-influx from intracellular stores [26]. ONOO^−^-activated Ca^2+^ uptake into sarco(endo)plasmic reticulum is mediated by SERCA through its S-glutathionylation at Cys674 [18], [19]. Permanent inactivation of SERCA by ThG could cause the deleterious effect of uncontrolled increases in intracellular Ca^2+^ through activation of apoptotic pathways [27]. Nitrosative stress caused by PABA/NO does not cause high levels of stable cysteine nitrosylation [3], but instead leads to S-glutathionylation of multiple intracellular proteins [6], [8]. Our present data show that these effects are also associated with an increase of cytosolic Ca^2+^, indirectly associated with PABA/NO-induced nitrosative stress. DETA/NO-mediated and DEA NONO-ate-mediated NO generation does not initiate Ca^2+^ flux, suggesting that direct NO is not mechanistically linked to the Ca^2+^ affect. This implicates the stable nitro-aromatic GSH-conjugate of PABA/NO as the likely inhibitor of SERCA [7]. Our results confirm that the kinetics of this reaction *in vitro* depend on the presence of the intracellular nucleophile GSH [7]. It is proposed that intracellular PABA/NO undergoes the same spontaneous or GST-catalyzed nucleophilic attack by GSH resulting in direct release of NO. However, the GSH-conjugated-nitro-aromatic metabolite shown in Fig. 8 is responsible for the indirect effects documented. Supporting this, our prior studies show that over-expressing MRP1 in mouse embryo fibroblasts conferred resistance to PABA/NO, suggesting that a toxic glutathione conjugate is effluxed from the cells [6]. In addition, the EC_50_ for ThG in tissue culture was in the range of 90–205 nM [28], [29]. Our data show ThG-mediated increase in NO generation with EC_50_∼85 nM. This similarity in EC_50_ is suggestive that SERCA-mediated intracellular Ca^2+^ fluxes are a likely reason for NO generation by eNOS.

HL60 cells share common progenitor stem cells with endothelial cells and other leucocytes. While both eNOS and iNOS are expressed in HL60 cells [30], [31], the former is significantly higher in HDMVEC. Our data show that NO response to the same dose of PABA/NO was ∼14 times higher in HDMVEC as compare to that in HL60 cells (Fig. 2, panel D). This indicates proportionality of PABA/NO-derived NO production to eNOS expression. Basal iNOS levels are stable for at least 72 h in HL60 cells [24]. We confirmed [31] that HL60 cells do not express NOS1. Depletion of eNOS (siRNA) or inhibition of eNOS or CaM (L-NAME, W-7) diminished NO generation and caused growth inhibition. Collectively, these data are compatible with a PABA/NO-induced and eNOS-mediated acute NO burst (∼10-fold increase) in HL60 cells. Similar but more pronounced PABA/NO effects were detected in HDMVEC.

PABA/NO treatment caused plasma membrane (***V_m_***) hyperpolarization/depolarization without an extracellular Ca^2+^ influx. HL60 cells express plasma membrane-associated NADPH oxidase [32] that can generate a superoxide radical (O_2_^-^). NO is ∼3 times more effective scavenger of O_2_^−^ than SOD [33] and this reaction produces ONOO^−^, an effective nitrosylating agent. The extracellular PABA/NO effect may be associated with S-nitrosylation of K^+^/Na^+^ channels, which corresponds to the cell surface cysteine modification presented in our present study. Similar observations of NO effects on ***V_m_*** in P388D.1 macrophage-like cells through K^+^ fluxes were reported earlier [34]. Additionally, ThG effects on ***V_m_*** were kinetically similar, but ∼10 times lower than those for PABA/NO, indicating a role for extracellular NO. By contrast, PABA did not induce any cell surface effects, confirming the importance of the NO-generating moiety. The drug-induced plasma membrane depolarization was diminished by eNOS depletion (data not shown). All these data indicate, not unexpectedly, that PABA/NO causes cell surface, as well as intracellular effects.

**Figure 9:**
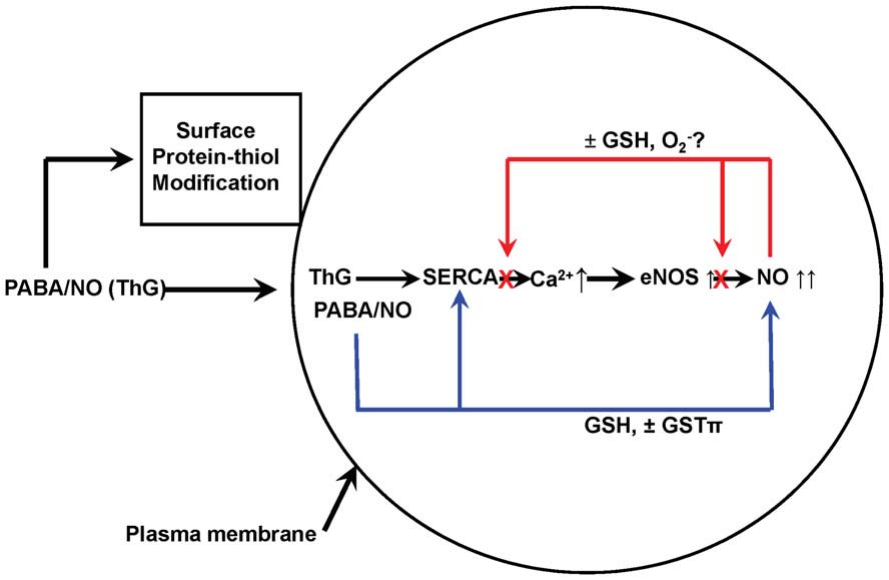
Scheme of PABA/NO effects on Ca^2+^/NO homeostasis in HL60 cells. PABA/NO effects start as extracellular NO-mediated (from spontaneous decomposition) surface protein-thiol modifications. Intracellular PABA/NO spontaneously (or under GSTP-catalysis) generates NO and stable nitro-aromatic compound(s) (PABA-SG). This can inhibit SERCA initiating a release of Ca^2+^ from the sarcoplasmic (endoplasmic) reticulum. The latter activates CaM and, consequently eNOS producing an NO burst. Increases of NO above threshold levels result in eNOS inhibition through its S-nitrosylation/glutathionylation. Once NO levels start to decline, spontaneous or catalyzed deglutathionylation of eNOS results in its reactivation to maintain steady state NO levels.

The active form of eNOS is a homodimer with zinc ions tetrahedrally coordinated to two pairs of symmetrical cysteines (Cys94 and Cys99 in each monomer [35], [36]). These cysteine residues are in a basic environment and as a consequence, have a low pK and may be subject to S-glutathionylation. It has been shown that nitrosylation of some of these cysteines results in dissociation of homodimers into inactive monomers [37]. eNOS is palmitoylated and therefore attached to an inner leaf of the plasma membrane. Thus, its activation results in an NO burst close to the plasma membrane. As well as an NADPH oxidase, there is a chloride ion channel-3 (CIC-3) [38] in the plasma membrane of HL60 cells. The Ca^2+^ fluxes could activate NADPH oxidase [39] and superoxide-radical generated outside the cells could influx through CIC-3 channels [40]. When spatially close, the eNOS and CIC-3 channels may generate ONOO^−^, which together with excess GSH can induce eNOS S-glutathionylation. The effect of PABA/NO on Ca^2+^/NO homeostasis in HL60 cells starts as an extracellular NO-mediated surface protein-thiol modification. Intracellular PABA/NO, spontaneously or under GSTP-catalysis, generates NO and the stable nitro-aromatic GSH-conjugate (PABA-SG) according to the scheme:
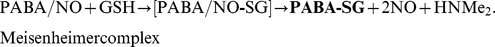



In our present study we showed that this metabolite inhibits SERCA, most likely, at the same site as ThG initiating an intracellular Ca^2+^ increase, activating CaM and consequently eNOS with the resultant NO burst. We hypothesize that PABA/NO causes intracellular NO levels to rise above a certain threshold through eNOS activation with a subsequent link between S-nitrosylation and S-glutathionylation. Indeed, there is evidence to suggest that two distinct pools of S-nitrosylated proteins exist, one that GSH stable and another that is GSH labile and subject to rapid conversion to a S-glutathionylated product [41], [42]. Both of these protein post-translational modifications have a role in signal transduction [43]. There are two mechanisms of NO-mediated protein S-glutathionylation: through a GSNO (activated thiol) formation and its consequent reaction with protein-thiol [44] or through an intermediate protein-thiol nitrosylation (activated protein-thiol: analogue of sulfenic acid) and its consequent reaction with the most abundant intracellular redox buffer, GSH [43]. The exact mechanism of eNOS modification is unknown but *in vivo* experiments have shown that eNOS activation in aortas and iNOS transgenetic expression in mouse heart both result in NO-induced protein S-glutathionylation [45]. Our present data are compatible with the interpretation that PABA/NO-mediated eNOS activation results in its S-glutathionylation in HL60 and HDMVE cells. This dynamic modification may serve to physiologically down regulate eNOS by NO under normal conditions [46]. Conversely, eNOS deglutathionylation via thioredoxin/thioredoxin reductase, glutaredoxin/glutaredoxin reductase [47], [48] or sulfiredoxin [14], [49], results in eNOS up-regulation, maintaining physiological NO levels. Under normal physiological conditions the NO increase might be controlled by S-nitrosylation/glutathionylation of eNOS as an immediate response or by similar modification/activation of SERCA in steady-state regulation (Fig. 9). Alternatively, in tumor cells with high levels of GSTP expression, the rate of PABA/NO-mediated NO increase could be substantially faster, outcompeting eNOS down-regulation through S-nitrosylation/glutathionylation and this could contribute to the cytotoxicity of the drug.

### Conclusions

Overall, the data are consistent with the model scheme in Fig. 9. The diazeniumdiolate has membrane effects that are attributable to NO release extracellularly or at the level of the plasma membrane. Intracellulary, the major nitroaromatic glutathione conjugate, primarily localized to the non-soluble cell fraction, can inhibit SERCA, increasing intracellular Ca^2+^ release and having an effect upon NOS that stimulates more NO release. NOS is subject to auto-regulation through S-glutathionylation. Thus, the drug has both a direct and indirect impact upon NO homeostasis and generates a chain of events that can alter cell-signaling pathways in a complex and inter-related manner.

## Materials and Methods

### Chemicals

Fura-2-AM (Acetoxymethyl 2-[5-[bis[(acetoxymethoxy-oxo-methyl)methyl]amino]-4-[2-[2-[bis[(acetoxymethoxy-oxo-methyl)methyl]amino]-5-methyl-phenoxy]ethoxy]benzofuran- 2-yl]oxazole-5-carboxylate), Fluo-3-AM (1-[amino-5-(2,7-dichloro-6-acetomethoxy-3-oxo-3*H*-xanthen-9-yl)phenoxy]-2-(2′-amino-5′-methylphenoxy)ethane-N,N,N′,N′-tetraacetic acid, penta-acetoxy-methyl ester), DAF-FM-DA (3-amino, 4-aminomethyl-2′,7′-difluorofluorescein diacetate), BAPTA-AM (1,2-bis-(o-Aminophenoxy)-ethane-N,N,N′,N′-tetraacetic acid, tetraacetoxymethyl ester), JC-9 (DiNOC1(3), 3,3′-dimethyl-a-naphthoxacarbocyanine iodide) and Bis-Oxonol (bis-(1,3-dibutylbarbituric acid)trimethine oxonol (DiBAC_4_(3)) were purchased from Invitrogen (Carlsbad, CA). ThioGlo® 1 (TG1, 3H-naphthol [2,1-b] pyran-s-carboxylic Acid; 10-(2,5-dihydro-2,5-dioxo-1H-pyrrol-1-yl)-9-methoxy-3-oxo-, methyl Ester) and Thapsigargin (ThG) were purchased from Calbiochem (San Diego, CA). PABA (4-aminobenzoic acid sodium salt), TCEP (tris(2-carboxyethyl)phosphine), W-7 (N-(6-aminohexyl)-5-chloro-1-naphthalenesulphonamide), 2,2′-(Hydroxynitrosohydrazono)bis-ethanimine (DETA/NO), and EGTA were purchased from Sigma (St. Louis, MO). L-NAME (*N^ω^*-nitro-L-arginine methyl ester) and DEA NONO-ate (Diethylammonium (Z)-1-(N,N-diethylamino)diazen-1-ium-1,2-diolate) were purchased from Cayman Chemical Company (Ann Arbor, MI). PABA/NO was a gift from Dr. Larry Keefer from the Chemistry Section, Laboratory of Comparative Carcinogenesis, NCI at Frederick (Maryland).

### Tissue culture

The human promyelocytic leukemia cell line HL60 was obtained from the American Type Culture Collection (ATCC; Rockville, MD) and maintained in RPMI-1640 with 10% heat-inactivated fetal calf serum (FCS) in a 5% CO_2_ atmosphere at 37°C. Primary culture of human dermal microvascular endothelial cells (HDMEC, [50]) and of human umbilical vein endothelial cells (HUVEC) were provided by Drs. S. Lenna and M. Markiewicz (both from the Department of Medicine, Division of Rheumatology of the Medical University of South Carolina). Cell number and viability were determined by trypan blue exclusion using Cellometer™ Auto T4 (Nexcelom, Biosciences, Lawrence, MA). Micoplasma was not detected in our cell cultures.

### Calcium labeling

Suspensions of HL60, TReNOS^−/−^ cells (1.0×10^6^cells/ml) and HDMVEC (∼90% confluence on Aclar plastic slides [51]) were incubated with 5 µM Fura-2-AM or Fluo-3-AM for 1 hour in complete media at 37°C. Labeled cells were washed 3 times with PBS, containing 100 µM CaCl_2_ or 4 mM EGTA, and used for experiments. To study the effect of chelating intracellular Ca^2+^, cells were incubated with 5 µM BAPTA-AM for 30 min before the addition of Fluo-3, and after 45 min of incubation were washed with PBS, containing 100 µM CaCl_2_ or 4 mM EGTA. PABA/NO was added to the specified final concentration of the drug (to avoid DMSO effects, the added volume never exceeded 5 µl). Viability of the intact, fluorescent-labeled control and PABA/NO-treated cells was measured for each experiment.

### Nitric oxide labeling

Suspensions of HL60, TReNOS^−/−^ cells (1.0×10^6^cells/ml) and HDMVEC (HUVEC) (∼90% confluence on Aclar plastic slides [51]) were incubated with 5 µM of DAF-FM-DA for 1 hour in complete media at 37°C. The labeled cells were washed 3 times with PBS, containing 100 µM CaCl_2_ or 4 mM EGTA.

### Kinetics of Calcium and NO

The dynamics of intracellular Ca^2+^ and NO changes were measured using a Modulus™ Microplate Multimode Reader (Turner BioSystems, Sunnyvale, CA) with a “Blue” optical kit (Ex = 490 nm, Em = 510–570 nm) and a standard kinetics mode. The measurements were performed at room temperature in 96-well Fluotrac 200 black microplates (VWR, Greiner Bio-One North America, Inc. Monroe, NC). The starting point for the kinetic experiments began ∼2 min after the addition of PABA/NO (e.g. the time necessary for the addition of drug to all wells).

Precise kinetic experiments were performed with the use of PTI QM-8 (PTI Inc., Birmingham, NJ), Hitachi F-2500 (Hitachi, Japan) and PTI QM-4 (PTI Inc.) spectrofluorometers and time-based fluorescence detection (resolution 0.1 sec). These experiments were performed in a 10×10×40 mm quartz cuvette under permanent stirring and temperature control. In these experiments, fluorescence was recorded for 100 sec prior and for indicated time after PABA/NO, DEA NONOate, DETA/NO, or ThG addition. Fresh prepared stock solutions of PABA/NO (100 mM in DMSO), DEA NONOate (100 mM in 0.01N NaOH), DETA/NO (100 mM in 10 mM PB, pH = 7.4), ThG (100 µM in DMSO) and PABA (100 mM in water, pH adjusted to 7.0) were used. All fluorescent measurements were corrected using the following controls: fluorescence of unlabeled cells, fluorescence changes in labeled cells without any addition of effectors and the effect of the addition of corresponding solvents to the fluorescence of labeled and intact cells.

### Total intracellular NO level in HDMVE and HL60 cells

In order to calculate intracellular NO concentration in HDMVEC, we used HUVEC as a standard. Both types of cells were grown on Aclar (Electron Microscopy Sciences, Hatfield, PA) plastic slides (12×20 mm) to ∼90% confluence under standard conditions in 30 mm Petry dishes. Cells were exposed to 5 µM of DAF-FM-DA in complete media for 1 h at 37°C and washed three times with PBS - to eliminate extracellular dye. A slide with the labeled cells was placed in a quartz cuvette (10×10×40 mm, containing 2.5 ml of PBS with 5 mM EGTA, pH = 7.4 at 37°C) under a 45° angle to the excitation beam, and the emission at 515 nm (excitation at 488 nm) was recorded for ∼5 min. After the measurement, the cells on the slide were trypsinized and counted using Cellometer™ Auto T4. The total slide area was 240 mm^2^ and the area from which fluorescence was acquired was ∼38.4 mm^2^ (for excitation and emission slits 2 and 4 nm, respectively). Thus the number of cells from which fluorescence was acquired, was (N_FL_ = N*0.9*(34.8/240)), where N – corresponds to the total number of cells, and 0.9 corresponds to the 90% confluence). The slides with DAF-FM-labeled HUVE and HDMVE cells were imaged under fluorescent microscope to confirm localization of fluorescence only inside cells (the level of labeling was >90%, data not shown).

For HL60 cells (suspension) labeling with DAF-FM-DA was the same as for the adherent cells. After washing them three times with PBS, 2 ml of labeled HL60 cells (1.0*10^6^ cells/ml) in PBS (100 µM of CaCl_2_ or 5 mM EGTA, pH = 7.4, 37°C) was placed in a quartz cuvette and the fluorescence of DAF-FM was recorded for 5 min. The labeled HL60 cells were also imaged under fluorescent microscope to show that fluorescence was localized inside the cells (the level of labeling was >90%, data not shown). The actual volume of the rectangular cuboid (under our experimental conditions) from which fluorescence was acquired was 64 mm^3^. The number of cells in this volume was 64*10^3^. The fluorescence of HUVEC or HDMVEC (5 slides of each) and HL60 cells (6 independent experiments) were averaged and normalized for cell number. The results of this analysis are presented in Table 1. The resting NO level in HUVEC was reported as 20 nM [52] and corresponds to a certain DAF-FM fluorescence per cell (Table 1). Thus the NO levels in HDMVE and HL60 cells are proportional to DAF-FM fluorescence per cell and could be calculated (Table 1).

### ThG, PABA/NO, and nitroaromatic compound (MW ∼622 Da) competition analysis

The Fluo-3-labeled (5 µM) HL60 cells (1.0*10^6^ cells/ml) were incubated with the indicated amount of PABA/NO or purified homogeneous nitroaromatic compound (MW ∼622 Da) for 20 min in PBS complemented with either 100 µM of CaCl_2_ or 5 mM of EGTA in a quartz cuvette at 37°C under constant stirring. After recording the background emission for 1 min, 100 nM (final concentration) of ThG was added to cell suspension and fluorescence was recorded for ∼5 min. The resulting kinetic curves were integrated using standard PTI software and normalized for ThG effect without the PABA/NO addition.

### Plasma membrane potential measurements

Suspension of HL60 cells (1.0×10^6^ cells/ml) in PBS with 100 µM CaCl_2_ (pH = 7.4) was incubated under constant stirring in a quartz cuvette at 37°C. The BisOxanol fluorescent probe in DMSO was added to cell suspension (final concentration 5 µM) and the kinetics of emission at 520 nm (Ex = 488 nm) were monitored before and after PABA/NO (ThG, PABA, DETA/NO) addition with a resolution of 0.1 sec for 5 min. Representative trace of three independent experiments was averaged and smoothed using standard SigmaPlot 10.0 (Systat Software Inc., San Jose, CA) software.

### Analysis of intracellular reduced protein thiols

Low molecular weight compounds were removed from the cell lysate using size-exclusion chromatography with a Bio-Spin 6 column (BioRad, Hercules, CA). After measuring protein concentration (Bradford, BioRad, Hercules, CA), the cell lysate was diluted 1∶100 with 20 mM sodium phosphate, pH = 7.4 in a quartz cuvette under constant stirring at 37°C. After the addition of TG-1 (5 µM), the kinetics of fluorescence at 513 nm (excitation at 379 nm) were recorded for ∼4 min. To measure surface thiol status in HL60 cells, TG-1 (final concentration 5 µM) was added to cell suspensions (1.0*10^6^ cells/ml) in PBS at 4°C under constant stirring, and kinetics of emission was recorded. The saturation fluorescence values of TG-1 treated cell lysates were normalized for protein content and cell surface thiol contents were normalized for the cell number and averaged using standard SigmaPlot 10.0 software (Systat Software Inc., San Jose, CA). At the end of each experiment, 1 µM GSH was added to the sample to ensure that the saturation of fluorescence was not caused by TG-1 content.

### siRNA-mediated inhibition of eNOS expression in HL60 cells

NOS3 siRNA(h) was purchased from Santa Cruz Biotechnology (Santa Cruz, CA). HL60 cells were transfected with NOS3 siRNA(h) to generate TReNOS^−/−^ cells according to the manufacturer's recommendation. Approximately 80% transfection efficiency was determined by flow cytometry with the use of FITC-labeled NOS3 siRNA(h) and confirmed by fluorescent microscopy (Nikon eclipse E800, Nikon Instr., Inc, Lewisville, TX with Nikon DS-U1 software v. 5.03, Photometrics, Tucson, AZ).

### Immunoprecipitation

Monoclonal antibodies against S-glutationylated proteins (Virogen, Watertown, MA) were conjugated with CNBr-activated sepharose (GE Healthcare) according to the manufacturer's recommendations. The yield of conjugation was controlled by measuring absorbency of antibody solution at 280/220 nm before and after conjugation and was ∼60–80%. After passing through a BioSpin-6 (BioRad) microcolumn (to eliminate low molecular weight thiols) HDMVEC lysates (∼200 µg of total protein) were incubated with a sepharose-conjugated antibody for 4–6 hours at room temperature under constant agitation. The sepharose immunoprecipitate was separated by centrifugation (16.0×10^3^ g, 5 min). After washing with PBS (3 times), the precipitated proteins were separated from the sepharose-conjugated antibody by acidification with glycine buffer (0.5 M, pH = 2.1) and collected by centrifugation (16.0×10^3^ g, 15 min, 4°C). The supernatants were concentrated using a CentriVap concentrator (LABCONCO Co, Kansas City, MO) and used for SDS PAGE and immunoblot analysis.

### Immunoblot analysis

HL60, TReNOS^−/−^ and HDMVE cells were lysed and cell homogenates were separated by centrifugation. Lysate protein was electrophoretically resolved by SDS-PAGE (8–10% Tris-Gly gel, 1 mm), and transferred to PVDF membranes (BioRad, Hercules, CA). Non-specific binding was reduced by incubating the membrane in a blocking buffer (20 mM Tris-HCl, pH 7.5, 150 mM NaCl, 0.1% Tween 20, 1 µM protease inhibitors, 5 mM NaF, and 1 mM Na_3_VO_4_) containing 10% non-fat dried milk for 1 hour. Endothelial NOS was immunostained with anti-NOS3 polyclonal antibody (1∶250), (C-20: Santa Cruz Biotechnology, Inc., Santa Cruz, CA) and detected with an HRP-conjugated secondary antibody and ECL (GE Healthcare Bio-Sciences Corp., Piscataway, NJ). eNOS electrophoresis standard (Cayman Chemical Co., Ann Arbor, MI) was used as a positive control for detection. The blots were scanned and visualized with a ChemiDoc system (BioRad). The relative intensity of bands was evaluated using Quantity One software (ver. 4.5.2; Bio-Rad) and plotted as arbitrary units (a.u.).

An infrared excited dual color (red and green) fluorescent imaging system (Odyssey LI-COR, NE) was used to detect S-glutathionylation of eNOS (eNOS-SSG) in HL60 cells after treatment with the indicated amounts of PABA/NO. Proteins from cell lysates were resolved electrophoretically and transferred to the PVDF membranes, as described above. Membranes were treated with the proprietary blocking buffer (LI-COR, NE) and incubated with both polyclonal anti-NOS3 and monoclonal anti-glutathionylated protein antibodies (1∶1,000 dilution, Virogen, Watertown, MA), as described above. After a 3× washing with PBS, an immunoblot was developed simultaneously with anti-rabbit IRDye®800CW (green) and anti-mouse IRDye®680 (red) secondary antibodies (1∶1,000 dilution, both from LI-COR, NE), as per manufacturer's protocol. Finally, immunoblots were imaged with an Odyssey imaging system (LI-COR) and quantified with standard software (LI-COR, NE).

### HPLC analysis

HPLC analysis was performed using 1525 binary pump, 2487 UV detector and 717plus Autosampler and Sunfire™ C18, 5 µm, 4.6×150 mm column (All from Waters, Milford, MA) with mobile phase ACN-0.1% formic acid (flow rate 1.0 ml/min) and detection at 315 nm. The mobile phase consisted of 25% ACN and 75%, 0.1% formic acid for 5 min, followed by a linear gradient to 75% ACN and 25% of 0.1% formic acid for the next 10 min. The PABA/NO (25 µM) reaction with GSH (2.0 mM) in 20 mM PB (pH = 7.4) was performed at room temperature and after incubation for the indicated time the samples were analyzed by HPLC. Absorbance and fluorescence spectra of the reaction mixture were also monitored. The purified PABA/NO metabolite (MW 622 Da) under our experimental conditions had a retention time of 16.849 min, absorbance spectra with maxima at ∼222 nm (compatible with specific absorbance of peptide bond) and 315 nm (corresponding with reported earlier specific absorbance for PABA/NO), and fluorescence spectra with emission maximum at 350 nm (excitation at 290 nm). ESI MS analysis of this purified product show singular most abundant (∼90% homogeneity) peak with M/Z = 623.8. To monitor metabolic conversion of PABA/NO we incubated HL60 cells (10.0*10^6^) with PABA/NO (50 µM) for 30 min at 37°C. Incubated cells were washed 3 times with PBS and lysed with standard cell extraction buffer (Invitrogen) with addition of a standard mixture of proteinase inhibitors (Sigma). After incubation of the cells with lysis buffer for 25 min at 4°C with periodic vortexing, samples were centrifuged at 16.1*10^3^ g for 5 min. The supernatant was collected and after addition of 10% (final concentration) of TCA and was again centrifuged at 16.2*10^3^ g for 15 min. The final supernatant was used for HPLC analysis. After primary HPLC analysis the peak with retention time of 16.84 min was identified. The addition of purified from *in vitro* reaction mixture PABA/NO metabolite (MW 622 Da) to this sample of cell lysate (“spike”) resulted in specific ∼2-fold increase of the peak at 16.84 min. Such data are consistent with the intracellular generation of this PABA/NO metabolite (MW 622 Da). Incubation of HL60 cells with purified nitro-aromatic compound (MW 622 Da, 15 µM, 35 min) resulted in its ESI MS detection in cell lysate after TCA precipitation. All HPLC data were processed using MPower software (built 2154, Waters Corp.).

### Mass spectrometric analysis

Samples were analyzed via liquid chromatography (LC)-electrospray ionization (ESI) -tandem mass spectrometry (MS/MS) on a linear ion trap mass spectrometer (LTQ, Thermo Finnigan) coupled to an LC Packings nano LC system. A 75micron C-18 reversed phase LC column (Microtech Scientific) was utilized with a 30 minute gradient from 2% acetonitrile, 0.2% formic acid to 60% acetonitrile, 0.2% formic acid. Data Dependant Analysis was utilized on the LTQ to perform MS/MS on all ions above an ion count of 1000. Dynamic Exclusion was set to exclude ions from MSMS selection for 3 minutes after being selected 2 times in a 30 second window.

### Statistical analysis of experimental data

All experimental data were statistically evaluated using SigmaStat 3.5 (Systat Software Inc., San Jose, CA) and represented as a mean±SEM (SD). The ANOVA was used to evaluate the relevance of a difference between control and treatment groups.
